# Can experienced surgeons predict the additional value of a CT scan in patients with displaced intra-articular distal radius fractures?

**DOI:** 10.1007/s11751-017-0283-9

**Published:** 2017-04-24

**Authors:** Y. V. Kleinlugtenbelt, K. Madden, S. R. Groen, S. J. Ham, P. Kloen, R. Haverlag, M. P. Simons, M. Bhandari, J. C. Goslings, V. A. B. Scholtes, R. W. Poolman

**Affiliations:** 1 Department of Orthopaedic and Trauma Surgery, Joint Research OLVG, Oosterpark 9, P.O. Box 95500, 1090 HM Amsterdam, The Netherlands; 2 Department of Orthopaedic and Trauma Surgery, Deventer Ziekenhuis, Nico Bolkesteinlaan 75, 7416 SE Deventer, The Netherlands; 3 Division of Orthopaedic Surgery, McMaster University, 293 Wellington St N Suite 110, Hamilton, ON L8L 8E7 Canada; 4 Department of Orthopaedic and Trauma Surgery, Academic Medical Centre, P.O. Box 22660, 1100 DD Amsterdam, The Netherlands; 5 Department of General and Trauma Surgery, OLVG, Oosterpark 9, P.O. Box 95500, 1090 HM Amsterdam, The Netherlands; 6 Trauma Unit, Department of Surgery, Academic Medical Center, University of Amsterdam, P.O. Box 22660, 1100 DD Amsterdam, The Netherlands

**Keywords:** Distal radius fractures, CT scans, Clinical decision making

## Abstract

There are no clear guidelines when an additional CT scan should be obtained for the treatment of displaced intra-articular distal radius fractures (DRF). This study aimed to investigate whether surgeons can predict the usefulness of CT scans to facilitate choice of treatment plan and/or pre-operative planning for DRF. Four surgeons evaluated 51 patients with displaced DRF. The choice of treatment (operative or nonoperative) was based on conventional radiographs. Subsequently, the surgeons were asked whether they would have requested an additional CT scan to determine this treatment choice, and also whether they required a CT scan for pre-operative planning. After 4 weeks, the additional CT scan was provided and the cases were assessed again. Based on these data, we calculated the number needed to scan (NNS) and number needed to harm (NNH) for two decision models. Model 1: Only provide a CT scan if the surgeon requested one based on their judgment of the X-rays. Model 2: CT scans for all displaced intra-articular DRF. For choice of treatment, the NNS was lower for model 1 than for model 2 (2.6 vs. 4.3) and the NNH is higher for model 1 (3.1 vs. 1.3). For pre-operative planning, the NNS (1.3 vs. 1.4) and NNH (3.7 vs. 3.4) were comparable for both models. Surgeons are able to predict the usefulness of an additional CT scan for intra-articular displaced DRF for OR indication. However, for pre-operative planning the usefulness of a CT scan is much harder to predict.

## Introduction

Multiple studies have shown an increased number of computed tomography (CT) requested by physicians [1–3]. This issue has become a subject of concern for patients, health care providers and regulators, and is receiving increasing attention in the medical literature [4]. CT scans are more expensive than X-rays, and national health care budgets are limited.

Recently, the liberal use of CT scans in the management of distal radius fractures (DRF), specifically for displaced intra-articular fractures, became widely accepted as an additional imaging tool in pre-operative evaluation and planning [5]. This increased popularity is supported by the previous literature. CT scans have been shown to be more reliable than X-rays in quantifying articular surface incongruencies [6–10]. Furthermore, when a treatment plan is based on both X-ray and CT scan, a surgeon is more likely to treat the DRF patient surgically than when the treatment plan is based on X-ray alone [11].

Does this mean that we should request a CT scan for all patients with displaced intra-articular fractures? Much information required for treatment planning can be obtained from plain X-rays. However, CT scans can give additional information which is not always seen on the X-ray. In DRF management, CT scans can be requested by the surgeon for two reasons: firstly, to decide whether to treat the patient operatively or nonoperatively, and secondly, for pre-operative planning purposes. Guidelines (e.g., AAOS, Dutch guidelines) have been developed to aid surgeons in decision making, but they are not clear about when to use an additional CT scan for the treatment of DRF [12]. Some surgeons have a low threshold to request a CT scan for displaced intra-articular DRF, while others rarely obtain a CT scan.

The aim of this study was to investigate whether surgeons can predict the usefulness of a CT scan in patients with displaced intra-articular DRF. This was done by comparing two decision models for when to request an additional CT scan.

## Methods

### Experimental design

In our experiment, we compared two different models of decision making.

Model 1: Only provide a CT scan if the surgeon requested one based on their judgment of the X-rays.

Model 2: CT scans for all displaced intra-articular DRF.

Moreover, we investigated this for two reasons for requesting an additional CT scan: (1) to decide whether to treat the patient operatively or nonoperatively (OR indication); and (2) for pre-operative planning (OR preparation).

### Patient selection

Consecutive patients with displaced distal radius fractures were selected from our Emergency Department database. The protocol in the recruiting department is to always order a CT scan for patients with a displaced intra-articular distal radius fracture. Patients were eligible for inclusion if they (1) presented with a displaced DRF in the Emergency Department between January 1, 2007 and March 2, 2011, (2) were 18 years of age or older, (3) had no prior fracture or pathology of the distal radius, (4) had both pre- and post-reduction plain posterior–anterior and lateral radiographs of the wrist and (5) had an additional post-reduction CT taken within 5 days after the reduction.

### Observers

Four experienced surgeons (two trauma surgeons and two orthopedic surgeons) reviewed the images. They all have over 10 years of experience in fracture treatment. All of them are responsible for the distal radius fracture care within their department.

### Time points

All surgeons scored the images at four different time points (T1–T4). Each scoring round was performed with an interval of at least 4 weeks.

T1 and T2: pre- and post-reduction plain radiographs.

T3 and T4: pre- and post-reduction plain radiographs and axial, sagittal and coronal planes CT. All images were digitized and anonymized, and presented with the relevant clinical data (e.g., age of the patient, gender, dominant hand, profession and specific hobbies).

### Scoring form

We used two scoring forms with the following questions.

T1 and T2:

1. Type of fracture: intra- or extra-articular.
2. Choice of treatment plan: nonoperative treatment with plaster after closed reduction or operative treatment (OR indication).
3. Would you request a CT scan for OR indication?: Yes or No?
4. If treated operatively, would you request a CT scan for pre-operative planning (OR preparation)?: Yes or No.

T3 and T4:

1. Choice of treatment plan: nonoperative treatment with plaster after closed reduction or operative treatment (OR indication).
2. Was the CT scan useful for OR preparation?: Yes or No.

### Methods to prevent bias

Surgeons were not informed that we were testing two decisions models and were blinded to the study hypotheses. We informed them that they were participating in an inter-observer reliability study. The order of the images was randomized to differ at all time points. Cases were presented in random order at different time points to prevent recall bias.

### Statistical analysis

We compared across time points with X-ray only (T1 and T2) and X-ray + CT scan (T3 and T4) which gives a total of four comparisons: T1–T3, T1–T4, T2–T3 and T2–T4. Therefore, we have 204 observations (4 surgeons; 51 cases) per comparison and 816 observations (204 observations; 4 comparisons) in total for our statistical analysis.

In both models of decision making, we assessed the number needed to scan (NNS) and number needed to harm (NNH) separately for the two reasons a CT scan is ordered (OR indication and OR preparation). NNS is akin to number needed to treat (NNT) for treatment studies. It is the number of patients who need a CT scan to achieve one additional good outcome. When requested for OR indication, a change in treatment (*Tx* change) is defined as a good outcome. When requested for OR preparation, useful for operative planning is defined as a good outcome. NNH is the number of patients on average need to be exposed to a risk factor to cause harm in an average of one patient who would not otherwise have been harmed. We defined two risk factors which could harm the patient. (1) Unnecessary radiation: Requesting a CT scan unnecessarily can harm a patient due to excess radiation. (2) Surgeon is suboptimally informed: Not requesting a CT scan when in fact the CT scan would have been useful can harm the patient because the surgeon is suboptimally informed about the fracture. The total NNH and the NNH for the two risk factors are determined separately. A low NNS and a high NNH are preferred.

In clinical practice, when a CT scan is ordered and the decision is made to treat the patient operatively based on the CT scan, it is not necessary to predict whether we need a CT scan for operative planning, because the CT scan is already available. We conducted a sensitivity analysis in which only patients who were treated operatively but who did not have a CT scan ordered for OR indication were included in the analysis.

Formulas for NNS/NNH.

Model 1:

$${\text{NNS}}_{\text{OR\,indication}} = \frac{1}{{(Tx\,{\text{change}}\,{\text{when}}\,{\text{CT}}\,{\text{requested}})/({\text{Total}}\,{\text{CT}}\,{\text{requested}})}}$$

$${\text{NNH}}_{\text{OR\,indication}} = \frac{1}{{({\text{No}}\,tx\,{\text{change}}\,{\text{when}}\,{\text{CT}}\,{\text{requested}}) + (Tx\,{\text{change}}\,{\text{when}}\,{\text{CT}}\,{\text{not}}\,{\text{requested}})/({\text{All}}\,{\text{observations}})}}$$

$${\text{NNS}}_{\text{OR\,preparation}} = \frac{1}{{({\text{CT}}\,{\text{useful}}\,{\text{when}}\,{\text{CT}}\,{\text{requested}})/({\text{Total}}\,{\text{CT}}\,{\text{requested}})}}$$

$${\text{NNH}}_{\text{OR\,preparation}} = \frac{1}{{({\text{CT}}\,{\text{not}}\,{\text{useful}}\,{\text{when}}\,{\text{CT}}\,{\text{requested}}) + ({\text{CT}}\,{\text{useful}}\,{\text{when}}\,{\text{CT}}\,{\text{not}}\,{\text{requested}})/({\text{All}}\,{\text{operative}}\,{\text{observations}})}}$$

Model 2:

$${\text{NNS}}_{\text{OR\,indication}} = \frac{1}{({Tx\,{\text{change}}\,{\text{when}}\,{\text{CT}}\,{\text{requested}}) + (Tx\,{\text{change}}\,{\text{when}}\,{\text{CT}}\,{\text{not}}\,{\text{requested}})/({\text{All}}\,{\text{observations}})}}$$

$${\text{NNH}}_{\text{OR\,indication}} = \frac{1}{{({\text{No}}\,tx\,{\text{change}}\,{\text{when}}\,{\text{CT}}\,{\text{requested}}) + ({\text{No}}\,tx\,{\text{change}}\,{\text{when}}\,{\text{CT}}\,{\text{not}}\,{\text{requested}})/({\text{All}}\,{\text{observations}})}}$$

$${\text{NNS}}_{\text{OR\,preparation}} = \frac{1}{{({\text{CT}}\,{\text{useful}}\,{\text{when}}\,{\text{CT}}\,{\text{requested}}) + ({\text{CT}}\,{\text{useful}}\,{\text{when}}\,{\text{CT}}\,{\text{not}}\,{\text{requested}})/({\text{All}}\,{\text{operative}}\,{\text{observations}})}}$$

$${\text{NNH}}_{\text{OR\,preparation}} = \frac{1}{{({\text{CT}}\,{\text{not}}\,{\text{useful}}\,{\text{when}}\,{\text{CT}}\,{\text{requested}}) + ({\text{CT}}\,{\text{not}}\,{\text{useful}}\,{\text{when}}\,{\text{CT}}\,{\text{not}}\,{\text{requested}})/({\text{All}}\,{\text{operative}}\,{\text{observations}})}}$$

We used SPSS version 22 to conduct these analyses.

## Results

### Patient characteristics

**Fig. 1:**
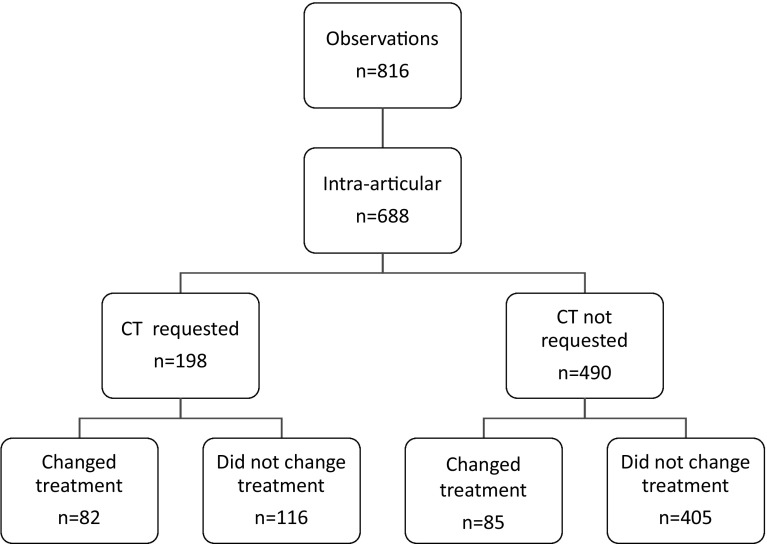
OR indication flowchart. Total score of the 4 observers

**Fig. 2:**
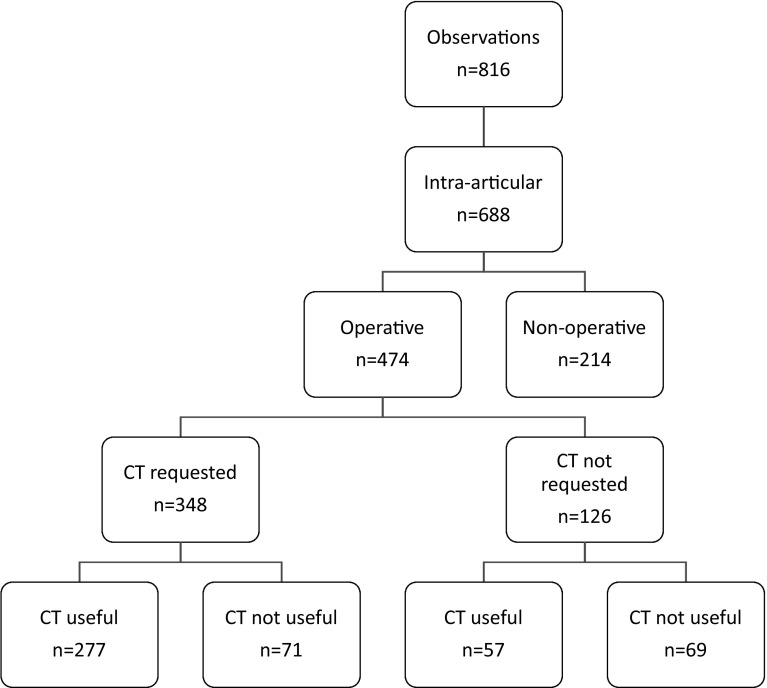
OR preparation flowchart. Total score of the 4 observers

During the study period, 85 patients who entered the Emergency Department with a displaced DRF had a post-reduction CT scan. A total of 51 patients met the complete inclusion criteria. Their mean age was 50 years (SD 14). Seventy-five percent of the patients were female. The CT scan was performed a mean of 2.53 days post-reduction (SD 2.21). Out of 816 observations, 688 times they were scored as intra-articular fractures (Figs. 1 and 2) and were included in this study.

### CT scan for OR indication

Of the 688 intra-articular observations based on X-rays only, the surgeon requested a CT scan for OR indication in 198 cases (198/688, 28.8%) (Fig. 1). For the cases where the surgeon requested a CT scan, the surgeon changed their treatment plan 41.4% of the time (82/198) with an additional CT scan. For the cases where the surgeon did not request a CT scan, the surgeon changed their treatment plan 17.3% of the time (85/490) with an additional CT scan. The NNS was lower for model 1 than for model 2 (2.6 vs. 4.3), and the NNH is higher for model 1 (3.1 vs. 1.3) (Table 1).

**Table 1:** CT scan for OR indication (with 95% CI)

	**NNS**	**NNH total**	*NNH: Suboptimal informed*	*NNH: Unnecessary radiation*
Model 1 (CT on request)	**2.4** (0.42)	**3.4** (0.39)	*8.1 (0.78)*	*5.9 (1.04)*
Model 2 (CT for all)	**4.1** (0.30)	**1.3** (0.03)	*0*	*1.3 (0.03)*

Total NNH values are in bold and those of the two subgroups are in italic

### CT scan for OR preparation

 In 474 observations, the surgeon decided to treat the patient operatively based on X-rays. The surgeon requested a CT scan for OR preparation in 348 cases (348/474, 73%) (Fig. 2). For the cases where the surgeon requested a CT scan for OR preparation, the CT scan was useful 80% of the time (277/348). For the cases where the surgeon did not request a CT scan, the CT scan was useful 45% of the time (57/126). The NNS (1.3 vs. 1.4) and NNH (3.7 vs. 3.4) are comparable for both models (Table 2). In the sensitivity analysis, which more closely approximates a real clinical situation, the NNS (1.2 vs. 1.3) and NNH (4.3 vs. 4.8) only slightly changed (Table 2).

**Table 2:** CT scan for OR preparation (with 95% CI) (Corrected: sensitivity analysis for clinical practice)

	**NNS**	**NNH total**	*NNH: Suboptimal informed*	*NNH: Unnecessary radiation*
Model 1 (CT on request)	**1.3** (0.02)	**3.7** (0.26)	*8.3 (1.2)*	*6.7 (0.35)*
Model 2 (CT for all)	**1.4** (0.06)	**3.4** (0.36)	*0*	*3.4 (0.36)*
Model 1 corrected	**1.2** (0.02)	**4.3** (0.42)	10.2 (1.21)	*7.6 (0.67)*
Model 2 corrected	**1.3** (0.03)	**4.8** (0.27)	0	*4.8 (0.27)*

Total NNH values are in bold and those of the two subgroups are in italic

## Discussion

### Main outcomes

Surgeons were able to predict the usefulness of an additional CT scan for intra-articular displaced DRF regarding whether one will advise the patient to be treated operatively or nonoperatively. The NNS for OR indication was clearly lower when the surgeons predict that the additional CT scan will be useful. The NNH was also lower, which means that when surgeons predict the usefulness of an additional CT scan, fewer CT scans are needed and therefore fewer patients are harmed than when surgeons order CT scans for all patients with intra-articular displaced DRF. The only disadvantage is that in one out of eight patients (NNH: 8.1) the surgeon is suboptimally informed compared to if they had had an additional CT scan.

However, for pre-operative planning the usefulness is much harder to predict, so it is more defendable to request CT scans for all intra-articular displaced DRF which are operatively treated. The NNS and NNH in each model are similar, even when corrected for the clinical practice. To choose the appropriate decision-making model for OR preparation, surgeons must weigh the pros and cons of each model based on which harm they think is most important to avoid. If surgeons request an additional CT scan for all operatively treated DRFs, there will be no patients for which the surgeon is suboptimally informed, but one in 3.4 patients will be exposed to radiation unnecessarily. If surgeons predict that an additional CT scan would be useful for OR preparation, the surgeon will be suboptimally informed in one out of 10 patients, but in only one out of 8 cases the patient will be exposed to radiation unnecessarily. They should also take into account the extra costs, which are about € 250 per scan in the Netherlands, of ordering additional CT scans in all cases.

### Strength and limitations

This study has several strengths including the use of highly experienced observers. These observers are typical surgeons who would make such decisions in hospitals, thereby improving generalizability. Also, the order of images was randomized and the time in between scoring moments was adequate to avoid bias due to memory. Surgeons were not informed we were testing two decisions models and were blinded to the study hypotheses. There is a potential risk of clustering; however, since the intra-observer agreement of classifying fractures and for treatment planning is known to be fair to moderate, we are justified in combining results of the four comparisons. Additionally, the relatively small confidence intervals of NNS and NNH show that the results are consistent across comparison groups. We used a database, so there is a possibility that in some less severe intra-articular displaced DRF, an additional CT scan was not ordered. The chance that a CT scan would have changed treatment plan in these cases is very low. However, the protocol in the recruiting hospital is to always order a CT scan for operatively treated cases, so this limitation would not have affected the results for OR preparation.

Although the previous literature showed that CT scans are more reliable than X-rays quantifying articular surface incongruencies [7–10, 13], to the best of our knowledge no previous studies have reported whether the usefulness of an additional CT scan is predictable.

### Future research

The outcomes of the current study are not necessarily related to better patient outcomes. Prospective randomized studies—comparing ordering CT scans for all patients with intra-articular displaced DRF to CT scanning on request of the surgeon—should be conducted to confirm the results of this study and to follow up patients to determine which decision-making model improves patient outcomes. Also a cost effectiveness analysis should be conducted as national care budgets are limited.

## Conclusion

Surgeons are able to predict the usefulness of an additional CT scan for intra-articular displaced DRF regarding whether one treats the patient operatively or nonoperatively. We recommend letting the surgeon decide which patients require an additional CT scan for treatment planning. However, for pre-operative planning the usefulness is much harder to predict, and therefore we cannot give a strong recommendation for or against CT scanning of all patients with a displaced intra-articular DRF.
